# Influence of Banana Genotype on Polyphenol Content and Starch Digestibility of Unripe Flours

**DOI:** 10.3390/foods14234048

**Published:** 2025-11-26

**Authors:** Edith Agama-Acevedo, Ana L. Solis-Mariano, Sherlin M. Altamirano-Monico, Vareska L. Zárate-Córdova, Luis A. Bello-Perez

**Affiliations:** 1 Instituto Politécnico Nacional (IPN), Centro de Desarrollo de Productos Bióticos (CEPROBI), Yautepec 62731, Morelos, Mexico; vreskazarate@csaegro.edu.mx (V.L.Z.-C.); labellop@ipn.mx (L.A.B.-P.); 2 Departamento de Ingenieria Bioquímica, Tecnológico Nacional de Mexico, Campus Acapulco, Acapulco 39905, Guerrero, Mexico; l20320533@acapulco.tecnm.mx (A.L.S.-M.); l20320360@acapulco.tecnm.mx (S.M.A.-M.)

**Keywords:** banana, dietary fiber, antioxidant capacity, starch hydrolysis, genotype

## Abstract

Banana fruits are consumed raw as desserts or after cooking. They are classified based on their genotype as pure (AAA) or hybrid cultivars (AAB). There is a growing interest in the use of unripe banana flours to produce functional foods due to the presence of polyphenols and dietary fiber (including resistant starch). This study aimed to investigate the impact of banana genotype on the content of polyphenols and starch digestibility. One pure cultivar and two hybrids (dessert and cooking cultivars) were studied. The chemical composition, polyphenol content, antioxidant activity, and starch hydrolysis rate were analyzed. The hybrid cultivars (manzano and plantain) showed a higher total starch content than the pure cultivar (morado); dietary fiber showed an inverse pattern, with the cooking hybrid cultivar (plantain) presenting the lowest value. The dessert hybrid (manzano) exhibited the lowest value of the FRAP radical, indicating the presence of different polyphenols in different banana genotypes. Rapidly digestible starch showed the highest value due to the cooking process, with the hybrid dessert banana (manzano) demonstrating the slowest starch digestion rate. The starch hydrolysis constant varied: morado presented the highest value, while the dessert hybrid (manzano) showed the lowest. These results can help to determine the applications of banana cultivars.

## 1. Introduction

There is a growing interest in the search for food crops with diversified end uses. Some tropical crops, such as bananas and mangos, are consumed in their ripe state. Large quantities are lost during fruit ripening for commercialization due to inadequate postharvest management. There is interest in producing unripe flours from banana cultivars of different genotypes to diversify their uses because bananas are an alternative source of indigestible carbohydrates. Unripe bananas, when consumed without cooking, are an important source of resistant starch (RS), a component of dietary fiber. Using the entire banana fruit offers an alternative to reducing the pollution problems generated by the final disposal of the peel after the pulp has been used to manufacture various foods, such as snacks and flour [1].

Banana crops are classified, based on consumption, into two categories: dessert and cooking [2]. Banana cultivars come from two wild types: *Musa acuminata* Colla (A genome) and *Musa balbisiana* Colla (B genome) [3]. The Musa cultivars exhibit different ploidy levels, resulting from the combination of A and B genomic haploids, which, in turn, form distinct subgroups. Dessert bananas are consumed when they reach the maturity stage, where starch is transformed into single sugars (fructose and glucose), which give them their sweetness. They include pure *M. acuminata* cultivars (AA, AAA, and AAAA) and some hybrid cultivars (AB, AAB, AAAB, ABB, and AABB) [4]. Cooking bananas are classified in the plantain group. They are processed using an oven, boiling water, and frying at different maturity levels. In their unripe state, where the starch content is approximately 70 g/100 g of the pulp (dry basis), the pulp or the whole fruit (pulp + peel) is used for flour preparation; this flour can be used as an ingredient to prepare bakery products [5].

Studies on unripe banana flours (pulp and pulp + peel) made from plantain reported high contents of indigestible carbohydrate, dietary fiber, and polyphenols. Using these banana flours in foods has functional effects, including an increased dietary fiber content, a reduced starch hydrolysis rate, and an enhanced antioxidant capacity [6]. Studies on flours made from banana cultivars of different genotypes are scarce. We hypothesized that differences in functional ingredients, such as the polyphenol content and starch hydrolysis rate (glucose liberation during digestion), in unripe banana flours are associated with genotype characteristics. This issue is crucial for determining banana cultivars with specific functional characteristics that are suitable for use in various foods.

## 2. Materials and Methods

### 2.1. Sample Collection and Sample Preparation

Unripe bananas from three varieties—morado (*Musa Acuminata*, Red Dhaka, *Musa* AAA, dessert type), manzano (*Musa* sp. (L.) AAB, dessert type), and plantain (*Musa Paradisiaca* L., Musa AAB, cooking type)—were obtained from a local farm in San Juan Bautista Tuxtepec, Oaxaca, at ripening state 1 (green) [7], between December 2024 and February 2025. This stage was determined based on the farmers’ expertise, considering external attributes such as peel color, fruit size, weight, and weeks of ripening.

The samples were cut without removing the peel (pulp + peel) and soaked in a 3% citric acid solution (*w*/*v*) to prevent enzymatic browning. The unripe banana slices were dried at 40 °C for 24 h. The dehydrated samples were subsequently ground and sieved through a 40-mesh screen (0.425 mm). The resulting flours were hermetically sealed in packaging and isolated from light. Two batches of each variety were prepared, and three repetitions of each batch were obtained.

### 2.2. Materials

The enzymes and agents utilized in the total starch analysis were sourced from Megazyme (K-RSTAR, Megazyme, Bray, Ireland). *Aspergillus niger* amyloglucosidase and porcine mucosa pancreatin were acquired from Sigma Aldrich (Sigma Chemical, St Louis, MO, USA), and calcium chloride dihydrate, sodium hydroxide, glacial acetic acid, and hydrochloric acid were acquired from Fermont (Monterrey, Mexico) for the starch hydrolysis kinetics analysis.

### 2.3. Chemical Composition

Proximal analysis of the unripe banana flours was carried out to determine the contents of macronutrients (ash, proteins (N × 5.85), fat, and carbohydrates) and moisture, following the AACC Approved Methods 08–01.01, 46–13.01, 30–25.01, and 44–15.02. Total dietary fiber was evaluated following the AACC Approved Method 32–21.01 [8]. Total starch content was quantified according to [9]. Soluble carbohydrates were calculated by difference (100 − [moisture + protein + lipid + ash + total dietary fiber + total starch]).

### 2.4. Extraction of Total Polyphenol Content

An aqueous–organic double extraction was performed following the procedure reported in [10]. In summary, 500 mg of unripe banana flour from each genotype was homogenized with 20 mL of acidified methanol (0.8 M HCl; 50:50 *v*/*v*) and agitated for 1 h. The mixture was then centrifuged at 2500 rpm for 25 min at 4 °C, after which the supernatant was collected. The remaining pellet underwent a second extraction using 20 mL of acetone (70:30 *v*/*v*), also under agitation for 1 h. Finally, both supernatants were pooled in a flask with a mixture of acidified methanol and acetone (50:50 *v*/*v*) and kept at 4 °C until further use.

### 2.5. Total Polyphenol Content

The total polyphenol content in the extracted supernatants was determined according to the method reported in [11]. Phenolic compounds were quantified using the Folin–Ciocalteu assay, and the results were expressed as milligrams of gallic acid equivalents per gram of dry sample.

### 2.6. Analysis of Antioxidant Capacity

#### 2.6.1. ABTS Assay

The 2,2′-azinobis-(3-ethylbenzothiazoline-6-sulfonic acid) radical (ABTS) assay was performed following the procedure of [12], with slight modifications. A stock solution was prepared by reacting 7 mM ABTS with 2.42 mM potassium persulfate and allowing the mixture to stand for 14 h at room temperature in the dark. Before use, the ABTS solution was diluted with phosphate buffer to obtain an absorbance of 0.70 ± 0.02 at 734 nm, as measured with a microplate reader. Subsequently, 20 μL of the aqueous–organic extract was combined with 280 μL of the diluted ABTS solution and incubated for 6 min. The absorbance was then recorded at 734 nm, and the results were expressed as μmol Trolox equivalents per gram of dry sample.

#### 2.6.2. DPPH Assay

Antioxidant capacity assays were adapted to a Thermo Scientific Multiskan Sky microplate reader (Thermo Fisher Scientific, Waltham, MA, USA). The reduction of the 2,2-diphenyl-1-picrylhydrazyl radical (DPPH) was evaluated according to the method of [13], with minor modifications. Briefly, 30 μL of the diluted sample was mixed with 270 μL of a DPPH solution (150 μmol/L) prepared in methanol–water (80:20, *v*/*v*) and homogenized for 60 s in a 96-well microplate. The mixture was then incubated for 40 min at room temperature in the dark, after which the absorbance was recorded at 515 nm. The antioxidant capacity was expressed as μmol Trolox equivalents per gram of dry sample.

#### 2.6.3. FRAP Assay

The ferric-reducing antioxidant power (FRAP) assay was carried out according to [14], with slight modifications, as described by [13]. The FRAP reagent was prepared by mixing acetate buffer (300 mM; pH 3.6), 2,4,6-tri(2-pyridyl)-s-triazine (TPTZ; 10 mM in 40 mM HCl), and ferric chloride (20 mM) in a 10:1:1 ratio (*v*/*v*/*v*). A 20 μL aliquot of the sample was combined with 280 μL of the FRAP reagent and incubated for 30 min at room temperature. The absorbance was then recorded at 593 nm, and the results were expressed as μmol Trolox equivalents per gram of dry sample.

### 2.7. In Vitro Starch Digestibility

This assay was performed following the procedure reported by [15]. Each unripe banana sample was weighed to the equivalent of 100 mg of starch. The samples were cooked at 100 °C for 15 min with continuous stirring and subsequently cooled to room temperature. Then, 4 mL of sodium acetate buffer (100 mM; pH 5) containing calcium chloride (5 mM) was added, followed by gentle vortex mixing.

The samples were incubated at 37 °C for 360 min. Subsequently, 1 mL of an enzymatic mixture consisting of pancreatin (8 UPS; 2.5 mg/mL) and amyloglucosidase (SIGMA, St. Louis, MO, USA, 300 U/mL; 4 μL/mL) was added. Aliquots of 100 μL were collected at 5, 10, 15, 20, 30, 40, 50, 60, 90, 120, 240, and 360 min. Each aliquot was centrifuged at 3000× *g* for 5 min, and the glucose content was determined using the GOPOD assay, Megazyme, International, Bray, Ireland. Measurements were performed with a Multiskan microplate reader after incubation at 37 °C for 10 min, recording the absorbance at 510 nm. Based on the results, three nutritionally relevant starch fractions were determined: rapidly digestible starch (RDS), slowly digestible starch (SDS), and resistant starch (RS). The starch fractions were determined using the following formulas:

(1) % glucose= (AT × Vt × C × D)/(As × Wt) × 100

where

AT = the absorbance of the test solution;

Vt = the total volume of the test solution;

C = the concentration (mg glucose/mL) of the standard;

As = the standard absorbance;

Wt = the weight in mg of the sample taken for analysis;

D = the dilution factor.

(2) AT = (GT − GL) × 0.9

(3) ADR = (G20 − GL) × 0.9

(4) ADL = (G120 − G20) × 0.9

(5) RS = AT − (ADR + ADL)

where

AT = total starch;

ADR (rapidly digestible starch) = glucose released in 20 min (G20) minus free glucose (GL), which represents starch that is digested quickly;

ADL (slowly digestible starch) = glucose released within 20 to 120 min;

RS (resistant starch) = starch that was not digested within 120 min.

The digestograms were fitted to the log-of-slope plot according to the following equation:

(6) C(t) = C∞ (1 − exp(−kHt))

(7) dC/dt = C∞ kH exp(−kHt)

(8) ln(dC/dt) = ln(C∞ kH) − kHt

A graph of ln(dC/dt) vs. t was obtained, where the slope is the starch digestion constant (k, min^−1^).

### 2.8. Statistical Analysis

The experimental data were expressed as means ± standard deviations (SDs). Statistical differences between the means were assessed using one-way ANOVA for proximal composition, total dietary fiber, total starch, total phenolic content, antioxidant capacity, and in vitro digestibility. Significance was considered at *p* ≤ 0.05.

## 3. Results and Discussion

### 3.1. Chemical Composition

The pure cultivar and the two hybrid cultivars presented a protein content ranging from 4.3 to 4.9 g/100 g flour on a dry basis (db), without significant differences (Table 1). Those protein values are associated with enzymes involved in the biosynthetic pathways, which are active during the fruit development stage. The lipid content was different in the analyzed cultivars; this method performed extraction with ether, and different substances (lipids, pigments, and other soluble compounds in ether) present in the sample were removed. Morado showed the highest value for lipid content, which can be attributed to the pigments (polyphenols) present in the peel, and no difference was found in the hybrid cultivars regarding chlorophyll. The ash content in the morado cultivar (pure cultivar) was slightly higher than in the hybrid cultivars. The ash content is related to the mineral substances present in the peel and pulp, and the pattern shows that the ash content depends on the genotype. The soluble carbohydrate content was different among the three cultivars analyzed: morado banana (pure cultivar) presented the highest value, while the manzano cultivar (hybrid) presented the lowest (Table 1). This result could be related to the development of the fruit, where diverse biosynthetic routes are involved and some routes are more active in different genotypes. The soluble carbohydrate content included single sugars, such as glucose, fructose, and sucrose. These sugars are actively involved in diverse biosynthetic routes during plant growth (in leaves, stems, roots, and fruits) and in the production of starch and non-starch polysaccharides in various developmental stages. The soluble carbohydrate content in unripe banana flour affects the functional and nutritional characteristics of the food produced from this raw material.

The total dietary fiber content, which includes non-starch polysaccharides, was high in the morado (pure) and manzano (hybrid) cultivars. The fruit peel in the flour is responsible for this observation because it contains cellulose, hemicellulose, and lignin, which are components of dietary fiber. It is essential to consider the banana genotype when using unripe banana flour as a food ingredient as it affects the dietary fiber content.

It is well known that unripe bananas are a source of starch, and it has been reported that uncooked unripe bananas are the richest source of resistant starch. A significant difference in total starch content was observed between the pure cultivar (morado) and the hybrid cultivars (plantain and manzano). Manzano showed the highest total starch content, and morado showed the lowest. The total starch content is crucial for the functional characteristics of products made from starchy raw materials, as it affects water retention and viscosity; it is also important from a nutritional perspective due to the caloric content of the product. The amylose/amylopectin ratio of banana starch from diverse varieties varied [16]. Starch with a high amylose content is associated with slower digestion [17].

The food matrix and processing are important factors in starch digestibility. The interaction between starch and other components (e.g., dietary fiber, proteins, and lipids) can form physical barriers or complexes that slow enzyme access. The higher dietary fiber in manzano compared with plantain could be a contributing factor [18].

### 3.2. Polyphenol Content and Antioxidant Capacity

Banana fruits are a source of polyphenols. The unripe flours of morado (pure) and plantain (hybrid) presented the highest total soluble polyphenols (TSPs). These values can be considered to be within the ranges reported for other fruits, such as buckthorn pomace, where a total polyphenol content (TPC) of around 11 mg gallic acid equivalents (GAE) was reported [19], orange (approximately 14 mg GAE/g), and Gala apple (9–10 mg GAE/g) [20]. Flavonoids, including catechin and quercetin, are present at low levels, and other polyphenols, such as gallic acid and hydroxybenzoic acid, are also present in the unripe banana flours. Despite their low levels, catechin and quercetin appear to play a predominant role in the antioxidant activity of unripe banana genotypes, as documented in earlier studies. This finding reinforces the well-established association between phenolic content and antioxidant capacity. Furthermore, prior studies have reported significant differences in the polyphenol content between different *Musa* spp. genotypes, suggesting an influence of genetic background on the phytochemical profile of unripe banana flours [4,21].

The antioxidant capacity of the unripe banana flours, determined by the three methods described above, showed a slight difference in the ABTS+ and DPPH assays. The DPPH assay showed the highest value, indicating that the polyphenols present in the banana flours have a higher affinity for this radical (Table 2). The manzano cultivar (hybrid) showed the lowest value for the FRAP radical out of the three cultivars. This observation indicates that different polyphenols are present in this banana flour, which can affect digestion [22].

The antioxidant capacity of polyphenols had been assessed in other fruits in previous research, such as Chinese date (72.11 ± 2.19 μmol Fe(II)/g), pomegranate (25.57 ± 0.53 μmol Fe(II)/g), guava (23.80 ± 1.44 μmol Fe(II)/g), sweetsop (22.04 ± 1.05 μmol Fe(II)/g), persimmon (16.97 ± 0.26 μmol Fe(II)/g), plum (sanhua) (14.79 ± 0.20 μmol Fe(II)/g), cherries (14.56 ± 0.33 μmol Fe(II)/g), pineapple (14.50 ± 0.06 μmol Fe(II)/g), Chinese wampee (14.20 ± 0.25 μmol Fe(II)/g), and mangosteen (0.11 ± 0.01 μmol Fe(II)/g) [23]; these fruits are all sources of polyphenols, but unripe banana flours have been found to have antioxidant capacity in addition to being a dietary fiber supply [24]. Furthermore, previous studies have revealed statistically significant differences between banana varieties in terms of total phenolic content and antioxidant capacity [25].

### 3.3. Starch Digestion Rate

**Figure 1:**
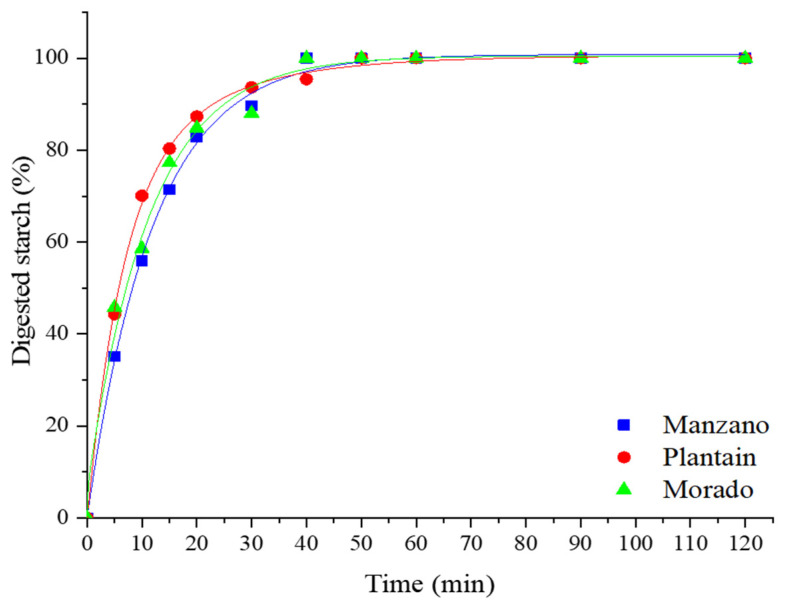
Hydrolysis rates of unripe banana flours. The graph was adjusted for the total starch content of the unripe banana flour from each genotype.

**Figure 2:**
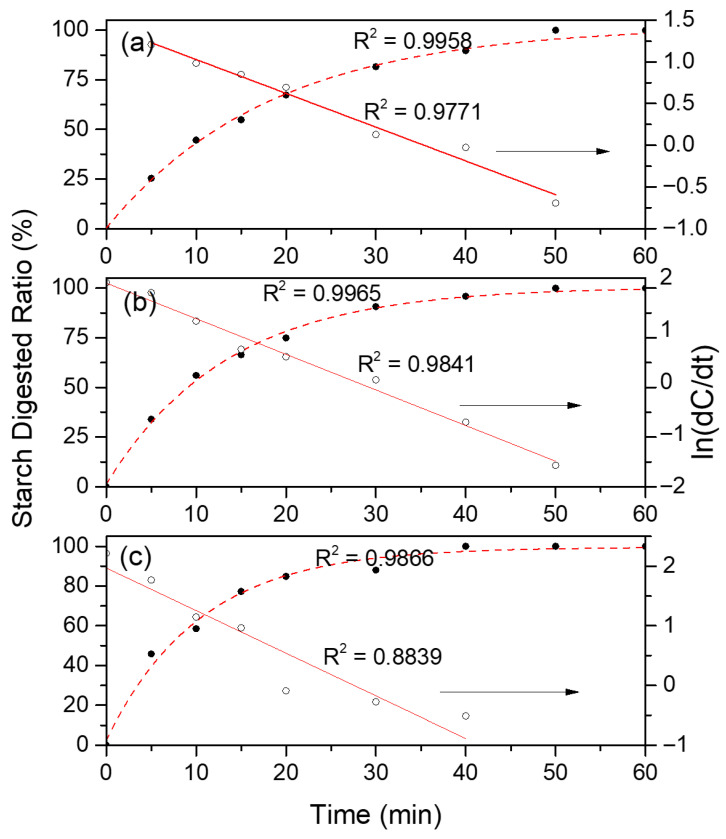
LOS plot based on the digestograms of unripe bananas from different genotypes: (**a**) manzano, (**b**) plantain, and (**c**) morado. The dotted line is experimental data, the continuous line is the mathematical fit.

The starch hydrolysis curves of cooked banana flours are shown in Figure 1. Unripe banana flour is used as a raw material in bakery products such as cookies, breakfast cereals, and pasta, which are typically consumed after cooking. The hybrid cultivars (manzano and plantain), which are consumed as dessert and after cooking, respectively, presented a very close pattern, while the pure cultivar (morado) was hydrolyzed at a higher rate. The three types of unripe banana flours exhibited a rapid hydrolysis rate in the first 50 min of the test, after which no further changes in glucose release were observed. This pattern occurred because the samples were cooked before the analysis, and starch became available for hydrolysis by digestive enzymes. Figure 2 shows a log-of-slope (LOS) plot of the hydrolysis kinetics of the three banana cultivars. The fit of the linear regression (R^2^ value) for the two hybrid cultivars was high, with morado showing the lowest value. These R^2^ values are considered acceptable for modeling the experimental data. The starch hydrolysis constants (Table 3) indicate different hydrolysis rates among the three cultivars, which is important for determining the applications of unripe bananas. These results align with Englyst’s test results, where the three fractions are rapidly digestible starch (RDS), hydrolyzed within 20 min; slowly digestible starch (SDS), hydrolyzed between 20 and 120 min; and resistant starch (RS), the residual fraction after a 120 min hydrolysis time. Table 3 shows the starch hydrolysis kinetic constants for the first phase (60 min) of the test estimated from the LOS plot. The pure cultivar (morado) presented the highest constant value. A difference in this value was observed for the hybrid cultivars, influenced by the consumption characteristic (dessert and cooking) of these cultivars; plantain (cooking) presented a higher constant value (faster) than the manzano cultivar (dessert). The LOS plot shows that the kinetic was achieved in one phase, with a correlation coefficient (R^2^) > 0.9 for manzano and plantain, and an R^2^ < 0.9 for the Morado cultivar. The banana cultivar presenting highest constant value is more susceptible to hydrolysis.

## 4. Conclusions

The morado cultivar showed the highest lipid content, which can be attributed to the soluble compounds in ether (polyphenols) present in the peel. No significant difference was found between the hybrid cultivars. The manzano (AAB) genotype, with its high total starch and slow digestion rate, is most suitable for developing foods with a low glycemic index. In contrast, the morado (AAA) genotype, with its high polyphenol content, may be better suited for enhancing the antioxidant capacity of foods. The whole fruit of morado (pure) and plantain (hybrid) presented the highest total soluble polyphenol (TSP) levels; this high concentration is related to the banana genotype, which affects the color peel and biosynthesis of bioactive compounds. Antioxidant capacity was not directly related to genotype. Some polyphenols present in the cultivars reacted differently with the radicals used in each antioxidant capacity method. The cooked fruit of the pure cultivar was hydrolyzed at a higher rate than the hybrid cultivars, with a higher difference observed in the slowly digestible starch content, which was not influenced by genotype. The obtained results can be used to determine the applications of banana cultivars in diverse foods to supply functional components, such as dietary fiber and polyphenols.

## Figures and Tables

**Table 1:** Proximate composition of whole unripe bananas of different genotypes (g/100 g DM).

Parameters	Manzano (AAB)	Plantain (AAB)	Morado (AAA)
Moisture	8.6 ± 0.1 ^a^	5.3 ± 0.1 ^c^	7.4 ± 0.4 ^b^
Protein	4.3 ± 0.2 ^b^	4.5 ± 0.2 ^b^	4.9 ± 0. 4 ^a^
Lipids	1.5 ± 0.2 ^b^	1.2 ± 0.0 ^b^	2.1 ± 0.0 ^a^
Ash	3.4 ± 0.1 ^b^	3.7 ± 0.0 ^b^	4.4 ± 0.2 ^a^
Soluble carbohydrates *	12.2 ± 0.3 ^c^	22.7 ± 0.2 ^b^	29.5 ± 0.4 ^a^
Total dietary fiber	12.8 ± 0.1 ^b^	8.0 ± 0.6 ^c^	15.4 ± 0.0 ^a^
Total starch	63.71 ± 1.7 ^a^	59.82 ± 0.8 ^a^	43.66 ± 1.9 ^b^

Values are reported as means ± standard deviations (*n* = 6). Mean values with different superscript letters within the same row are significantly different (*p* < 0.05). * Soluble carbohydrates were calculated by difference. Letters in brackets indicate the genotypes.

**Table 2:** Determination of polyphenol content and antioxidant capacity of whole unripe bananas of different genotypes.

		Manzano (AAB)	Plantain (AAB)	Morado (AAA)
Polyphenols	TSPs (mg GAE/g db)	5.36 ± 0.1 ^b^	6.25 ± 0.2 ^a^	6.78 ± 0.3 ^a^
	Flavonoids (mg CE/g db)	0.40 ± 0.02 ^a^	0.29 ± 0.03 ^b^	0.40 ± 0.04 ^a^
Antioxidant capacity	ABTS+ (µmol TE/g db)	6.20 ± 0.1 ^a^	5.21 ± 0.7 ^b^	5.07 ± 0.8 ^b^
	DPPH (µmol TE/g db)	52.79 ± 0.4 ^a^	47.78 ± 1.1 ^b^	54.97± 0.3 ^a^
	FRAP (µmol TE/g db)	6.33 ± 0.5 ^b^	17.30 ± 0.2 ^a^	17.36 ± 0.4 ^a^

Values are reported as means ± standard deviations (*n* = 6). Different letters in the same row indicate statistically significant differences (*p* < 0.05). db: dry basis; TSPs: total soluble phenolics of supernatant via aqueous organic extraction; ABTS+: 2,2′-azino-bis (3-ethylbenzothiazoline-6-sulfonic acid); DPPH: 2,2-diphenyl-1-picrylhydrazyl; FRAP: ferric-reducing antioxidant power; CE: catechin equivalents; GAE: gallic acid equivalents; TE: Trolox equivalents.

**Table 3:** Starch digestibility fractions of whole unripe bananas of different genotypes (%).

Sample	RDS	SDS	RS	K × 10^−2^ (min^−1^)
Manzano	74.59 ±0.37 ^c^	17.11 ± 0.41 ^a^	8.28 ± 0.04 ^c^	40.56 ± 0.253 ^c^
Plantain	78.56 ± 0.25 ^a^	12.71 ± 0.28 ^c^	8.72 ± 0.03 ^a^	68.38 ± 0.329 ^b^
Morado	76.32 ± 0.50 ^b^	15.18 ± 0.55 ^b^	8.48 ± 0.05 ^b^	71.46 ± 0.046 ^a^

Values are reported as means ± standard deviations (*n* = 6). Mean values followed by different letters in the same column indicate a significant difference (*p* < 0.05). RDS: rapidly digestible starch; SDS: slowly digestible starch; RS: resistant starch;  K is the starch hydrolysis constant.

## Data Availability

The original contributions presented in this study are included in the article. Further inquiries can be directed to the corresponding author.
